# The Uptake of Pneumococcal and Seasonal Influenza Vaccinations Based on Perceptions and Attitudes Toward the COVID-19 Vaccine Among Patients With Diabetes

**DOI:** 10.7759/cureus.56943

**Published:** 2024-03-26

**Authors:** Melike Mercan Baspinar, Arzu Demirali

**Affiliations:** 1 Family Medicine, Gaziosmanpaşa Taksim Training and Research Hospital, Istanbul, TUR; 2 Nursing, Gaziosmanpaşa Taksim Training and Research Hospital, Istanbul, TUR

**Keywords:** nursing, pneumococcus, influenza, diabetes, vaccination, covid-19

## Abstract

Objective

In this study, we aimed to assess the rates of pneumococcal and seasonal influenza vaccinations among elderly and nonelderly diabetes patients and examine their perceptions and attitudes toward the coronavirus disease 2019 (COVID-19) vaccine.

Methods

A single-center study was conducted among patients with diabetes, employing a structured survey encompassing sociodemographic data, vaccination records, and the COVID-19 vaccine perception and attitude scale.

Results

Among the 280 diabetes patients in our study, the vaccination rates for COVID-19, seasonal influenza, and pneumococcal vaccines were 96.1%, 16.8%, and 17.5%, respectively. A higher cumulative dosage of the COVID-19 vaccine was associated with older age (r = 0.463; p<0.001), increased safety score (r = 0.479; p<0.001), and lower conspiracy theory score (r = -0.336; p<0.001). Participants who had received COVID-19 and influenza vaccines were observed to have significantly higher safety scores related to COVID-19 vaccines (p<0.001; d = 2.381 and p = 0.008; d = 0.525, respectively). Notably, vaccination rates for influenza and pneumococcus were significantly different between nonelderly and elderly patients (8.7% vs. 29.6%; p<0.001 and 13.4% vs. 24.1%; p = 0.022). Elderly patients with diabetes were 3.3 times more likely to receive the influenza vaccine than nonelderly participants [odds ratio (OR) = 3.319; 95% confidence interval (CI) = 1.592 - 6.920; p = 0.001] and had a higher safety score related to COVID-19 vaccines (OR = 1.076; 95% CI = 1.011 - 1.146; p = 0.021).

Conclusions

Both influenza and pneumococcal vaccination rates were below the desired targets in this study. The vaccination rates among the nonelderly diabetes population suggest that this group may be more likely to neglect to receive vaccination compared to the elderly diabetes population. The association between vaccination rates and post-pandemic safety perceptions highlights the critical need to implement public health strategies specifically designed to address and improve safety-related information dissemination.

## Introduction

Health organizations and government authorities worldwide advocate for routine vaccination as a highly effective measure to mitigate the risk of influenza and invasive pneumococcal disease among both elderly and nonelderly high-risk patients [1]. The mortality rate associated with coronavirus disease 2019 (COVID-19) infection is as high as 17-38% in older patients with chronic diseases, with diabetes mellitus observed in 5.3-42.3% of fatalities [2].

Primarily, seasonal influenza vaccination reduces the risk of hospitalization and mortality in diabetic patients aged ≥65 years [3]. Secondly, Streptococcus pneumoniae is identified as the most common coinfecting pathogen in COVID-19 cases. Approximately half of the fatal COVID-19 cases exhibit pneumonia-related complications. Specifically, in patients aged >65 years, pneumonia-related COVID-19 mortality was observed to be 23% [4]. Notably, the Ministry of Health in Turkey incorporated all diabetic patients, irrespective of age, into its routine vaccination program for the dual pneumococcal vaccine (PCV13+PPV23) in 2016, in line with the guidelines published by the Ministry of Health and the Association of Endocrinology. Also, routine annual influenza vaccination is recommended for all individuals aged ≥6 months [5,6]. On the other hand, vaccination rates of adult influenza and pneumococcal vaccines remain low [6]. The TEMD Vaccination Study indicates that patients with diabetes in Turkey exhibit notably low influenza vaccination rates, with 23.6% in type 1 diabetes mellitus (T1DM) and 21.2% in type 2 diabetes mellitus (T2DM). Pneumococcal vaccination rates were even lower, at 8% in T1DM and 7% in T2DM patients [7].

Since the onset of the COVID-19 pandemic, vaccine confidence has markedly decreased [8]. This decline in confidence has been observed across all demographic groups, indicating a widespread reduction in trust in vaccines in the wake of the pandemic [8]. The experience of immunization against Ebola in various African nations has demonstrated that social and political opposition can emerge when new vaccinations are deployed during public health emergencies [9]. Therefore, effective immunization programs must disseminate adequate safety information to counter doubts and conspiracy theories [10]. Various international publications report different prevalence rates of adult vaccine administration in diabetic patients. However, data on this topic are scarce, especially during and after pandemics. Our study suggests that post-pandemic immunization rates for pneumococcal and seasonal influenza vaccines may vary among diabetic patients based on their perception and attitude toward the COVID-19 vaccine. We aimed to determine the relationship between vaccination rates and post-pandemic safety perceptions and conspiracy theories about COVID-19 vaccines.

This study was previously presented as a meeting abstract at the Second Black Sea Family Medicine Days in May 2023.

## Materials and methods

Study design

This observational study involved diabetic patients who had been referred from internal medicine and family medicine clinics to the diabetes education department of a tertiary hospital in Istanbul between April 30 and June 30, 2022. The sample size calculation aimed to reach out to all patients applying to attend diabetes education, with an anticipated two-monthly number of 500 adult patients, based on a 0.95 confidence level and 25% probable vaccination rate [11] with a 30% dropout rate factored in; the final manuscript reached 280 volunteers. All participants signed an informed consent form, agreed to participate, and could drop out at any stage. The study was approved by the Ethics Committee of Istanbul Provincial Health Directorate Gaziosmanpaşa Training and Research Hospital on April 13, 2022 (access number: 60).

Study instrument

The patients were asked to fill out specifically designed questionnaires involving sociodemographic characteristics (age, marital status, education, occupation, and income), concomitant diseases, prior COVID-19 history, and vaccination data. The history of pneumococcal vaccination after diagnosis of diabetes and influenza vaccination in the past year was obtained from the patient's statements, and the hospital's vaccination records confirmed it. Additionally, we assessed perceptions and attitudes towards the COVID-19 vaccine by using a specific scale.

The COVID-19 Vaccine Perception and Attitude Scale

This 5-point Likert scale consists of 21 expressions and two sub-dimensions; the perception of safety and the perception of conspiracy theory were developed by Eriş in 2021 [12]. Statements 7, 8, 10, and 11 in the scale are reverse coded. Statements in the scale are scored as follows: "I strongly agree = 5", "I agree = 4", "I am undecided = 3", "I disagree = 2", "I strongly disagree = 1". The scale's overall score is not computed, given the negative correlation between the subscales. Instead, as the score for the safety subscale rises, the score for the conspiracy theory subscale drops, leading to separate evaluations of each subscale. The reliability values for the scale's sub-dimensions used in the study were notably high, with the safety perception subscale achieving a reliability rate of 0.903 and the conspiracy theory perception subscale reaching a reliability rate of 0.891 [12].

Statistical analysis

Categorical variables were summarized using frequencies and percentages, whereas continuous variables were described using means and standard deviations (SD). To compare safety and conspiracy theory scores across study groups, the t-test was utilized. A p-value of less than 0.05 was considered statistically significant. The effect size of significant findings was determined using Cohen's d test. A commonly used interpretation involves referring to effect sizes as small (d = 0.2), medium (d = 0.5), and large (d = 0.8) based on benchmarks suggested by Cohen [13]. The internal consistency of the COVID-19 vaccine perception and attitude scale was assessed using the Cronbach alpha score, with a score above 0.7 considered high [14]. Correlations were analyzed using the Pearson correlation coefficient. Risk factors related to elderly diabetic patients versus others were evaluated with binary logistic test results. Data analysis was performed using the EPICOS statistic program.

## Results

A total of 280 participants diagnosed with diabetes mellitus, with an average age of 59.7 ± 13 years (range = 18-93 years, median = 62 years) were included in the study. Among the participants, 151 (53.9%) were female and 129 (46.1%) were male; 38.6% (n = 108) of the patients were aged over 65 years. Of note, 96.1% (n = 269), 17.5% (n = 49), and 16.8% (n = 47) were vaccinated with the COVID-19 vaccine, pneumococcal vaccine, and seasonal influenza vaccine, respectively; 47.5% (n = 133) had previously contracted COVID-19. Among the COVID-19 vaccines administered, 55.7% (n = 156) of the participants had received the Sinovac vaccine, 82.1% (n = 230) had received the BioNTech vaccine, and only 0.7% (n = 1) had received the Turkovac vaccine. The COVID-19 vaccine perception and attitude scale demonstrated high internal consistency in this study, with Cronbach's alpha values of 0.830 for the safety perception subscale and 0.816 for the conspiracy theories perception subscale.

Participant characteristics by age and gender are presented in Table 1. No gender differences were detected in terms of vaccination rates for COVID-19, pneumococcal, and influenza (p = 0.510, p = 0.259, p = 0.666, respectively). Older adults aged over 65 years were less likely to have had a prior COVID-19 infection compared to the working age group (p = 0.011). We found significant differences in vaccination rates between diabetes patients aged 18-65 and those over 65 years old for both influenza (8.7% vs. 29.6%; p<0.001) and pneumococcal vaccines (13.4% vs. 24.1%; p = 0.022).

**Table 1:** Evaluation of baseline characteristics of study participants according to age and gender groups P<0.05 is considered statistically significant COVID-19: coronavirus disease 2019

Variables	Groups	Total	Gender		P-value	Age group		P-value
		n (%)	Female (n = 151), n (%)	Male (n = 129), n (%)		18-65 years (n = 172), n (%)	≥65 years (n = 108), n (%)	
Education	Primary/secondary school	210 (75.0%)	123 (81.5%)	87 (67.4%)	0.007	118 (68.6%)	92 (75.0%)	0.002
	High school/university	70 (25.0%)	28 (18.5%)	42 (32.6%)		54 (31.4%)	16 (14,8%)	
Occupation	Not working	183 (65.4%)	134 (88.7%)	49 (38.0%)	0.001	92 (53.5%)	91 (84.3%)	<0.001
	Working	97 (34.6%)	17 (11.3%)	80 (62.0%)		80 (46.5%)	17 (15*7%)	
COVID-19 disease history	Positive	133 (47.5%)	72 (47.7%)	61 (47.3%)	0.947	92 (53,5%)	41 (38.0%)	0.011
	Negative	147 (52.5%)	79 (52.3%)	68 (52.7%)		80 (46.5%)	67 (62.0%)	
COVID-19 vaccination	Vaccinated	11 (3.9%)	144 (95.4%)	125 (96.9%)		163 (94.8%)	106 (98.1%)	0.156
	Unvaccinated	269 (96.1%)	7 (4,6%)	4 (3,1%)	0.51	9 (5.2%)	2 (1.9%)	
Pneumococcal vaccination	Vaccinated	49 (17.5%)	30 (19.9%)	19 (14.7%)	0.259	23 (13.4%)	26 (24.1%)	0.022
	Unvaccinated	231 (82.5%)	121 (80.1%)	110 (85.3%)		149 (86.6%)	82 (75.9%)	
Seasonal influenza vaccination	Vaccinated	47 (16.8%)	24 (15.9%)	23 (17.8%)	0.666	15 (8.7%)	32 (29.6%)	<0.001
	Unvaccinated	233 (83.2%)	127 (84.1%)	106 (82.2%)		157 (91.3%)	76 (70.4%)	

Table 2 illustrates the impact of receiving COVID-19, pneumococcal, and influenza vaccines on the perceptions toward safety and conspiracy theories related to the COVID-19 vaccine. Safety and conspiracy scores were compared between groupwise variables. Older adults over 65 years and females scored higher on the perception of conspiracy theories related to the COVID-19 vaccine (p = 0.015; Cohen’s d = 0.292 and p = 0.043; Cohen’s d = 0.242, respectively). Participants vaccinated against COVID-19 and seasonal influenza displayed significantly higher safety perception scores related to COVID-19 vaccines compared to those who were unvaccinated (p<0.001; Cohen’s d = 2.381 and p = 0.008; Cohen’s d = 0.525, respectively). Participants vaccinated against COVID-19 and seasonal influenza had lower scores for perception toward conspiracy theories. However, the effect size results from comparing mean scores between vaccinated and unvaccinated groups were not substantial enough to draw definitive conclusions (p<0.001; Cohen’s d = -1.745 and p = 0.002; Cohen’s d = -0.412, respectively).

**Table 2:** Comparison of age, sex, and vaccination status among participants based on perception toward safety and conspiracy theories related to COVID-19 vaccines P<0.05 is considered statistically significant. Cohen’s d: effect size classified as small (d = 0.2), medium (d = 0.5), and large (d = 0.8) COVID-19: coronavirus disease 2019; SD: standard deviation

Variables	Groups	Perception of safety related to COVID-19 vaccine (score), mean ± SD	P-value	Cohen’s d	Perception of conspiracy theories related to COVID-19 vaccine (score), mean ± SD	P-value	Cohen’s d
Age	18-65 years (n = 172)	35.83 ± 6.36	<0.001	-0.487	27.80 ± 5.31	0.015	0.292
	≥65 years (n = 108)	38.69 ± 4.93			26.32 ± 4.62		
Sex	Female (n = 151)	36.35 ± 6.02	0.079	-0.21	27.8 ± 5.10	0.043	0.242
	Male (n = 129)	37.61 ±5.94			26.57 ± 5.03		
COVID-19 vaccination	Vaccinated (n = 269)	37.40 ± 5.54	<0.001	2.381	26.96 ± 4.93	<0.001	-1.745
	Unvaccinated (n = 11)	25.64 ± 6.19			33.91 ± 4.85		
Pneumococcal vaccination	Vaccinated (n = 49)	37.29 ± 5.99	0.872	1.712	26.20 ± 6.11	0.111	-0.227
	Unvaccinated (n = 231)	36.86 ± 6.02			27.45 ± 4.84		
Seasonal influenza vaccination	Vaccinated (n = 47)	39.21 ± 4.13	0.008	0.525	25.28 ± 4.87	0.002	-0.412
	Unvaccinated (n = 233)	36.47 ± 6.22			27.63 ± 5.06		
COVID-19 disease history	Vaccinated (n = 133)	36.18 ± 6.16	0.048	-0.239	27.77 ± 5.01	0.09	0.203
	Unvaccinated (n = 147)	37.61 ± 5.80			26.74 ± 5.14		

As shown in Table 3, the analysis of pneumococcal and seasonal influenza vaccine vaccination rates revealed no significant difference between participants with and without COVID-19 vaccination (p = 0.454 and p = 0.129, respectively). It was observed that all individuals who had received the influenza vaccine, and all but one patient vaccinated against pneumococcal disease, had also received the COVID-19 vaccine, indicating a 100% overlap for the influenza vaccine recipients.

**Table 3:** Comparison of vaccination rates of pneumococcal or seasonal influenza vaccines between participants with and without COVID-19 vaccination P<0.05 is considered statistically significant. Cohen’s d: effect size classified as small (d = 0.2), medium (d = 0.5), and large (d = 0.8) COVID-19: coronavirus disease 2019

Variables	Groups	COVID-19 vaccination		P-value	Cohen’s d
		Vaccinated	Unvaccinated		
Seasonal influenza vaccination	Vaccinated	47 (17.5%)	0	0.129	0.877
	Unvaccinated	222 (82.5%)	11 (100.0%)		
Pneumococcal vaccination	Vaccinated	48 (17.8%)	1 (9.1%)	0.454	0.428
	Unvaccinated	221 (82.2%)	10 (90.9%)		

Table 4 depicts the correlations between the total number of COVID-19 vaccine doses received, age, and scores for safety perception and conspiracy theory perceptions related to the COVID-19 vaccine. A higher total number of COVID-19 vaccine doses was associated with an increased perception of safety (r = 0.479; p<0.001), older age (r = 0.463; p<0.001), and a decreased perception of conspiracy theories (r = -0.336; p<0.001).

**Table 4:** Evaluation of the correlation of total COVID-19 vaccine dosage and age with safety and conspiracy theory scores related to the COVID-19 vaccine P<0.05 is considered statistically significant; r = Pearson correlation test COVID-19: coronavirus disease 2019

		Total dose of COVID-19 vaccination	Perception of safety related to COVID-19 vaccine (score)	Conspiracy theory perception related to COVID-19 vaccine (score)	Age
Total dose of COVID-19 vaccination	r	1	-	-	-
	p	-	-	-	
Perception of safety related to COVID-19 vaccine (score)	r	0.479	1,000	-	-
	p	0	.	-	-
Conspiracy theory perception related to COVID-19 vaccine (score)	r	-0.336	-0.599	1,000	-
	p	0	0	-	-
Age	r	0.463	0.261	-0.192	1,000
	p	0	0	0.001	-

The risk factors of vaccination rate related to a diabetic patient aged ≥65 years versus others were assessed with a binary logistic regression model, and the results are shown in Table 5. The analysis revealed that a higher perception of safety score related to COVID-19 vaccines [odds ratio (OR) = 1.076; 95% confidence interval (CI) = 1.011 - 1.146; p = 0.021], receiving seasonal influenza vaccination (OR = 3.319; 95% CI = 1.592 - 6.920; p = 0.001), and a lower level of education (OR = 2.935; 95% CI = 1.464 - 5.887; p = 0.002) were significantly associated with being an elderly diabetic patient as opposed to other diabetic patients. This indicates that elderly diabetic patients are 3.3 times more likely to receive the influenza vaccine than their younger counterparts.

**Table 5:** Evaluation of factors related to diabetic patients aged ≥65 years versus those aged 18-65 years P<0.05 is considered statistically significant (binary logistic regression test) COVID-19: coronavirus disease 2019; OR: odds ratio; CI: confidence interval

Variables	OR	95% CI	P-value
Education (low)	2.935	1.464 – 5.887	0.002
Prior COVID-19 history	0.614	0.362 – 1.043	0.071
COVID-19 vaccination	0.829	0.149 – 4.624	0.831
Pneumococcal vaccination	1.629	0.795 – 3.341	0.183
Seasonal influenza vaccination	3.319	1.592 – 6.920	0.001
Perception of safety related to COVID-19 vaccine (score)	1.076	1.011 – 1.146	0.021
Conspiracy theory perception related to COVID-19 vaccine (score)	0.993	0.928 – 1.062	0.836

## Discussion

Distrust of vaccines and susceptibility to conspiracy theories have always existed in the general population; however, the degree to which patients' attitudes towards newly developed vaccines would affect other adult vaccination rates was unknown in the wake of the COVID-19 pandemic. Our study constitutes one of the novel studies investigating the associations of COVID-19 vaccination and perceptions related to it with the frequency of receiving influenza and pneumococcal vaccine among patients with diabetes during the COVID-19 pandemic. The vaccination rates for COVID-19, seasonal influenza, and pneumococcal disease were 96.1%, 16.8%, and 17.5%, respectively. A higher total dosage of the COVID-19 vaccine was associated with older age, increased safety scores, and decreased conspiracy theory scores. Additionally, patients aged over 65 years showed significantly higher rates of influenza and pneumococcal vaccinations than younger patients, with the elderly receiving the influenza vaccine 3.3 times more frequently. Despite higher vaccination rates among the elderly compared to those aged 18-65 years, the study highlights that influenza and pneumococcal vaccination rates remain significantly lower compared to the high rate of COVID-19 vaccination.

The World Health Organization recommends an influenza vaccination rate of at least 75% in high-risk populations [15]. In developing countries, the reported rates of influenza vaccination vary widely, ranging from as low as 0.4% to as high as 28%. Similarly, the rates for pneumococcal vaccination in these regions also show considerable variation, ranging from 0.7% to 26% [16]. In a pre-pandemic study conducted over several influenza seasons involving 241,551 patients with diabetes, the coverage rate for the influenza vaccine ranged from 24% to 36% [17]. Vaccination rates can vary across different patient demographics as well. In patients diagnosed with pneumoconiosis, the rates for receiving the influenza and pneumococcal vaccines were found to be 19.2% and 21.9%, respectively [18]. In individuals with chronic obstructive pulmonary disease (COPD) attending chest disease outpatient clinics, influenza and pneumococcal vaccination rates were found to be 40% and 10%, respectively. Additionally, among patients hospitalized in ICUs for community-acquired pneumonia, the rate of receiving both pneumococcal and influenza vaccines was observed to be 6% [19,20].

Preventive care services play a crucial role in preventing or delaying complications associated with diabetes [21]. The onset of the pandemic has led to uncertainties regarding vaccination. However, a study conducted in a family medicine center, comparing pre- and post-pandemic vaccination rates, found an increase in adult vaccination rates following the pandemic. Specifically, the rate of conjugated pneumococcal vaccination rose from 50.7% to 75.5%, and the rate for seasonal influenza vaccination increased from 1% to 13.1%, according to the records of patients included in the study [22]. As per one study, from 2008 to 2020, no improvement was observed in preventive care service use among U.S. adults with diabetes except for influenza vaccination [21]. According to the findings of the TEMD Vaccination Study, the rate of influenza vaccination among diabetic patients in Turkey is below 25%, and the rate of pneumococcal vaccination is below 10% [7]. In our study, while the rate of influenza vaccination was lower compared to the TEMD study results, the rate of pneumococcal vaccination was nearly twice as high.

The study by Yılmaz et al. demonstrated that a regimen of four doses of the COVID-19 vaccine, in combination with the influenza and pneumococcal vaccines, offered protection among elderly individuals living in nursing homes [23]. In the study conducted by Liang et al., among the participants who received the influenza vaccine, 60.7% had received at least one dose of the COVID-19 vaccine [24]. In our study, the COVID-19 vaccination rate was notably high at 96.1%, and among those who received the influenza vaccine, 100% had also received the COVID-19 vaccine. This pattern of high uptake was consistent with that of the pneumococcal vaccine, except for one patient in our study sample. Given these findings, establishing a routine adult vaccination program could meet expectations for accepting new vaccine types in future pandemics. Our results suggest that vaccine acceptance or rejection may be influenced by the general perception toward vaccinations rather than opinions on specific vaccine types.

In a pre-pandemic study of 350 diabetic patients, Yeşilova et al. observed that only 10.8% had received the pneumococcal vaccine, with a notable increase in vaccination rates associated with older age [16]. In our study, influenza and pneumococcal vaccine uptakes were higher in older adults in both genders. Consequently, the notably lower vaccination rates among the younger diabetic population compared to those of the elderly emerged as a critical finding of this study, suggesting a potential for higher hospitalization rates among the younger diabetic cohort. A study conducted in 2022-2023 revealed that high-risk populations show low intent to receive the influenza vaccine. This finding underscores the need to focus on addressing concerns related to the vaccine's safety and effectiveness, to encourage wider acceptance and uptake among these vulnerable groups [25].

Our findings showed that older age, higher safety scores, and lower conspiracy theory perceptions were related to higher total dose amounts of COVID-19 vaccines, similar to the rate of influenza vaccination. According to two novel meta-analyses, various sociodemographic factors, such as older age, higher education level, male gender, and ethnicity/race (e.g., Whites vs. African Americans), positively correlate with acceptance and uptake of COVID-19 vaccines [26]. A higher level of awareness and knowledge about vaccines, leading to higher safety perspectives and fewer doubts, was also associated with increased acceptance. Hesitancy towards vaccination was commonly attributed to concerns about safety and efficacy, a perception of low risk, the inconvenience of traveling long distances to vaccination centers, unfavorable vaccination schedules, trust in the healthcare system, public health authorities, and governments, and history of vaccination; vaccine-specific factors, and concerns about the rapid development of the vaccine [26,27]. In this study, conspiracy scores among diabetic women versus men were significantly higher but safety scores were similar. In light of our results, dynamics of increased safety perspective related to rapidly developed vaccines such as COVID-19 vaccines among elderly patients with diabetes, which contrast with the decreased safety scores among the nonelderly diabetic population, may be investigated in future studies.

Diabetes education provided by nursing staff specializing in diabetes care offers a valuable opportunity to remind diabetic patients about the importance of vaccinations and encourage those who have not yet been vaccinated to receive vaccines. A tele-nursing education study highlighted the effectiveness of such educational interventions, showing a significant increase in knowledge - from an average of 33.16% before the education to 71.72% after participating in the program, resulting in an average difference of 38.56%. This demonstrates the potential impact of targeted educational efforts on improving vaccine awareness and uptake among diabetic patients [28]. A study conducted in China examined the attitudes towards COVID-19 vaccination among diabetic patients and healthy individuals. The findings revealed a negative attitude and low level of awareness regarding vaccination among diabetic patients versus non-diabetics. The study suggests that by promoting knowledge and educating patients, social and medical workers can enhance the vaccination rates among diabetic individuals [29]. Another intervention project study about delivering influenza vaccinations in emergency departments has demonstrated that educating nurses was valuable in enhancing nurses’ knowledge and positive attitudes about providing influenza vaccines to patients [30]. In our study, all participants were drawn from the diabetes education nursing department. Diabetes care nursing and diabetes education for patients have a vital role to play in the follow-up of patients of all ages with diabetes; we recommend that the vaccination status of diabetes patients be included as a part of the diabetes care nursing discipline, which would have a positive impact on vaccine-related knowledge and safety perceptions.

This study has a few limitations, especially its cross-sectional design and the collection of the data based on self-reports and hospital data, which may have posed challenges in recalling whether the vaccination was administered, especially among older patients. Secondly, the diabetic patients included in the study were not classified as to whether they had type 1 or type 2 diabetes, the characteristics of which may differ from each other.

## Conclusions

Our findings reveal that both elderly and nonelderly patients with diabetes have insufficient influenza and pneumococcal vaccination rates when compared to COVID-19 vaccination rates. Positive associations of vaccination with increased safety scores and decreased conspiracy theory scores among vaccinated individuals have highlighted the need to educate the general population regarding perceptions of safety related to all vaccines. Ultimately, our study underscores the importance of coordination between diabetes care nurses responsible for patient education and physicians who manage treatment, to improve post-pandemic vaccination rates among diabetic patients. This collaborative approach could better address preventive health measures, including vaccination, as an integral part of diabetes management.
